# Remarks on Medical Reform

**Published:** 1813-11

**Authors:** 


*To the Editor of the Medical and Physical Journal.*

SIR,

THE bill introduced into parliament last sessions by certain apothecaries, but withdrawn by them, from a suspicion, perfectly well grounded, that the wisdom of parliament would reject it, is again to be presented in order to be passed into a law. As this bill is intended to produce changes of considerable moment and interest both to the public and the profession, a short and candid review of it may be useful and acceptable.

First, it divides England and Wales into a certain number of districts, to be governed by medical committees, which district committees are to be entirely subordinate to a certain new organised body called the *London Superintending Committee;* and in order to insure this subjection, the individual members composing the district committees are to be *appointed* by the London superintending committee.

The provincial committees are not to be permitted to examine or grant licenses to practitioners of any description, nor even to visiting assistants: this power is to be vested exclusively in the London committee.

The duty of the provincial committees is to be limited to the humble task of examining and binding apprentices, and granting licences to journeymen shopmen and female midwives, and faithfully report their proceedings, to be reviewed by the London committee. The fair inference from this regulation is, that the inferior attainments and professional knowledge of the gentlemen in the country disqualify them from passing judgment on the qualifications of candidates.

The district committees are to make out annually, regular lists of every licensed practitioner, and affix them to their respective parish church doors. The reason given for this is, to enable the public to distinguish the regulars from the irregulars. On this clause it may be remarked that few gentlemen of known and acknowledged reputation will permit their names to be enrolled or published in this manner. Besides the utility of it is very questionable; for when the advertising quack is consulted, it is not because he is mistaken for a regular, but because he is known to be a nostrum vender, and the avowed enemy of the regular practitioner.

All the money, says the bill, arising from certificates, is to be at the uncontrolled disposal of the London committee. With this fund they may purchase lands, build halls, endow teachers, and doubtless *grant to themselves adequate salaries for their great care and trouble.*

Such is the new medical constitution by which the profession are now to be governed. There remains to be noticed a few minor clauses or enactments which respect apprentices, female midwives, and attendance fees.

Apprentices, on being bound, are to be previously examined on their knowledge of the Greek Testament; their indentures are to bear a stamp duty of 25*l.*; and they are to pay a further sum of 5*l.* 5*s.*, which last sum is *to be shared by the London and the district committees.* We pass over the pedantic folly of requiring from apprentices a knowledge in the Greek Testament, to observe that such an enactment will effectually preclude every gentleman of narrow income and practice from bringing up his sons to his profession, and will drive multitudes into Scotland for their medical education, in which country apprentices will not be subjected to pay such unreasonable fees to a business, the emoluments of which are very often of the most slender and precarious nature.

Every female practising or acting as a midwife without a certificate, the bill enacts, is to be fined in a blank sum of money. By this clause it follows, should any good motherly woman assist another in the pains of labor, and take any reward for her services, she is to be punished by a fine. No exceptions are provided from the necessity of the case, or the absence of a licensed practitioner. This humane enactment may be fitly classed with the clause exacting a sum of money from the poor youth who hires himself as an assistant, "whose learning is probably all his portion," before he can be allowed to earn his bread; but, thank God, this rapacious bill is not yet passed into a law.

Apothecaries, the bill says, may sue for and recover fees for visits and attendance. The power of suing for and recovering such charges has never yet been denied the profession by either courts or juries. The writer of this article has himself witnessed, in the court of Chester, actions brought for such fees, within this year or two, and liberal verdicts given. In truth, this bill will add considerably to the expence of a process; for it enacts that no apothecary shall be allowed to recover *any charges* unless he shall prove himself duly licensed under this act.

But the most dangerous privilege which the London committee assume to themselves, is the power given to them by this act of making and revoking bye-laws, rules, and constitutions, as they see fit.

Upon the whole, it appears that this bill is in the highest degree objectionable, is unjust in its principle, and oppressive in its operation; and will prove only beneficial to the members of the London superintending committee:—to them them it will be a source of profit, power, and patronage; to the rest of the profession insulting and degrading. Besides, the creation of this new body is unnecessary and uncalled for: when the public service or government require a test of the professional abilities of any individual, previous to employing him, there exist already a College of Physicians and Surgeons, whose competency and integrity have never yet been questioned.

With respect to private practice, mankind have and will always claim the right of applying to whom they please for relief from their maladies. The scholar and well-educated practitioner, by a faithful discharge of his professional duties, will not fail in due time to evince his superiority over the unprincipled practitioner, "and holding thus the noiseless tenor of his way," will reach at length an honorable independence. MEDICUS STOCKPORTENSIS.*

*Sept.* 15, 1813.

* We are compelled to remark in common candor, that the observations in this paper, by *Medicus Stockportensis,* apply *only* to the bill which has been relinquished, and can have no reference to that which is intended to be brought into parliament in the ensuing session, the clauses of which not yet being before the public, cannot possibly be the subject of animadversion. As far as we are enabled to judge, from the *resolutions* which the Committee have printed, and which this writer might have seen in our preceding number, as the bases of the new bill, his remarks will be totally inapplicable to that bill.—EDITOR.

